# Accuracy of Computer-Aided Dynamic Navigation Compared to Computer-Aided Static Navigation for Dental Implant Placement: An In Vitro Study

**DOI:** 10.3390/jcm8122123

**Published:** 2019-12-02

**Authors:** Alfonso Mediavilla Guzmán, Elena Riad Deglow, Álvaro Zubizarreta-Macho, Rubén Agustín-Panadero, Sofía Hernández Montero

**Affiliations:** 1 Department of Implant Surgery, Faculty of Health Sciences, Alfonso X el Sabio University, 28691 Madrid, Spain; mediavillaalfonso@gmail.com (A.M.G.); elenariaddeglow@gmail.com (E.R.D.); shernmon@uax.es (S.H.M.); 2 Department of Stomatology, Faculty of Medicine and Dentistry, University of Valencia, 46010 Valencia, Spain; rubenagustinpanadero@gmail.com

**Keywords:** computer-assisted surgery, image-guided surgery, implantology, navigation system, real-time tracking

## Abstract

Aim: To analyze the accuracy capability of two computer-aided navigation procedures for dental implant placement. Materials and Methods: A total of 40 dental implants were selected, which were randomly distributed into two study groups, namely, group A, consisting of those implants that were placed using a computer-aided static navigation system (*n* = 20) (guided implant (GI)) and group B, consisting of those implants that were placed using a computer-aided dynamic navigation system (*n* = 20) (navigation implant (NI)). The placement of the implants from group A was performed using surgical templates that were designed using 3D implant-planning software based on preoperative cone-beam computed tomography (CBCT) and a 3D extraoral surface scan, and the placement of group B implants was planned and performed using the dynamic navigation system. After placing the dental implants, a second CBCT was performed and the degree of accuracy of the planning and placement of the implants was analyzed using therapeutic planning software and Student’s *t*-test. Results: The paired *t*-test revealed no statistically significant differences between GI and NI at the coronal (*p* = 0.6535) and apical (*p* = 0.9081) levels; however, statistically significant differences were observed between the angular deviations of GI and NI (*p* = 0.0272). Conclusion: Both computer-aided static and dynamic navigation procedures allow accurate implant placement.

## 1. Introduction

Dental implant placement has recently emerged as a predictable treatment option to restore edentulous patients [1]. Nevertheless, some complications are attributed to this technique, including cortical or dental perforations and damage to particular anatomical structures, such as the inferior alveolar nerve or the maxillary sinus, due to implant malpositioning [1,2]. Recently, dental implant placement using image data-based navigation has been introduced into the field of dental surgery in an attempt to improve the accuracy of dental implant placement and avoid potential risks associated with this therapeutic procedure [3]. This surgical approach was developed based on preoperative cone-beam computed tomography (CBCT) scanning and specific 3D implant-planning software, thereby allowing for accurate implant placement [4]. Generally speaking, there are two types of computer-assisted surgical implant placement system techniques, namely, static navigation and dynamic navigation. Static navigation systems require the use of surgical templates to guide the drilling process. Dynamic navigation systems recognize and track the position of optical reference markers placed over the patient and surgical instruments by means of a tracking system array. Both navigation techniques have been widely analyzed, demonstrating high accuracy levels for dental implant placement [1,3,5,6]. Static navigation systems have a mean horizontal deviation at the coronal entry point and apical endpoint of 1.2 mm (1.04–1.44 mm) and 1.4 mm (1.28–1.58 mm), respectively, and a mean angular deviation of 3.5° (3.0–3.96°) [7]. However, dynamic navigation systems demonstrated lower deviation values at the coronal entry point (0.71 ± 0.40 mm), apical endpoint (1.00 ± 0.49 mm), and angular deviation (2.26 ± 1.62°) [8], but these values have not yet been compared.

The aim of this work was to analyze and compare the accuracy of dental implant placement via static and dynamic navigation systems, with a null hypothesis (H0) stating that there would be no difference between the static and dynamic navigation systems with regard to the accuracy of dental implant placement.

## 2. Materials and Methods

### 2.1. Study Design

A randomized controlled experimental trial was conducted in accordance with the principles defined in the International Organization for Standardization (ISO 14801). The study was performed at the Dental Centre of Innovation and Advanced Specialties at Alfonso X El Sabio University (Madrid, Spain) between January and March 2019. This study was authorized by the Ethical Committee of the Faculty of Health Sciences, University Alfonso X el sabio, in December 2018.

### 2.2. Experimental Procedure

Forty dental implants (BioHorizons, Birmingham, AL, USA) were placed in tooth positions 2.4 and 2.6 (4.6 mm × 12 mm, conical wall and internal taper) in twenty standardized polyurethane models of partially edentulous upper jaws (Sawbones Europe AB, Malmo, Sweden) based on one obtained from a real clinical case. The dental implants were randomized (Epidat 4.1, Galicia, Spain) and assigned to one of two study groups: group A, consisting of dental implants that were placed using a computer-aided static navigation system (NemoStudio^®^, Nemotec, Madrid, Spain) (*n* = 20) (guided implant (GI)) and group B, consisting of dental implants that were placed using a computer-aided dynamic navigation system (Navident, ClaroNav, Toronto, Canada) (*n* = 20) (navigation implant (NI)). The dental implants were first placed in tooth position 2.4, followed by position 2.6, in consideration of a real situation.

**Figure 1:**
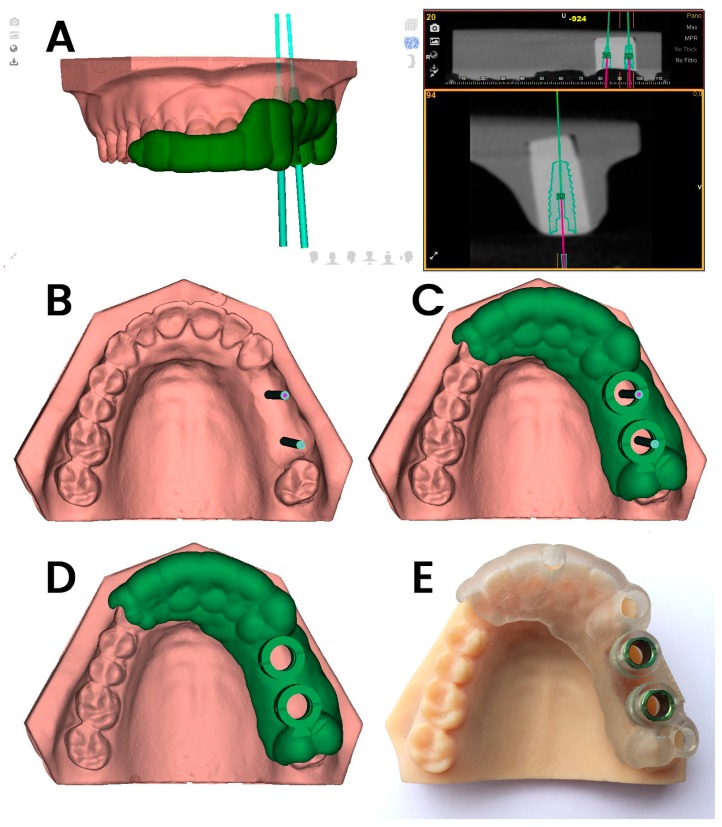
(**A**–**C**) Dental implant planning with the computer-aided static navigation system using a cone-beam computed tomography (CBCT) scan (green lines); (**C**,**D**) virtual template design according to the planned virtual dental implant placement; (**E**) manufactured stereolithography template fixed over the dental surface of the teeth and placed over the partially edentulous upper jaw models.

The GI jaw models were submitted for a preoperative CBCT scan (WhiteFox, Satelec, Merignac, France) with the following exposure parameters: 105.0 kV peak, 8.0 mA, 7.20 s, and a field of view of 15 mm × 13 mm. Afterward, the ten jaw models assigned to the GI study group were submitted for a 3D-extraoral surface scan (EVO, Ceratomic, Protechno, Girona, Spain). Datasets obtained from the digital workflow were uploaded into 3D implant-planning software (NemoStudio^®^) to design virtual templates for GI implant placement. The 3D surface scan and CBCT data were matched by aligning the key points present in the partially edentulous upper jaw models. A virtual implant drill was designed with a diameter and length of 4.6 and 12 mm, respectively, as per the measurements of the selected implant (Figure 1A,B). After designing the virtual templates (Figure 1C,D), they were fabricated using the stereolithography technique (ProJet 6000, 3D Systems, Rock Hill, SC, USA) (Figure 1E), except for the 5 mm stainless steel cylinders, which were manually attached to the templates. The templates fit the model and did not need further adjustments.

**Figure 2:**
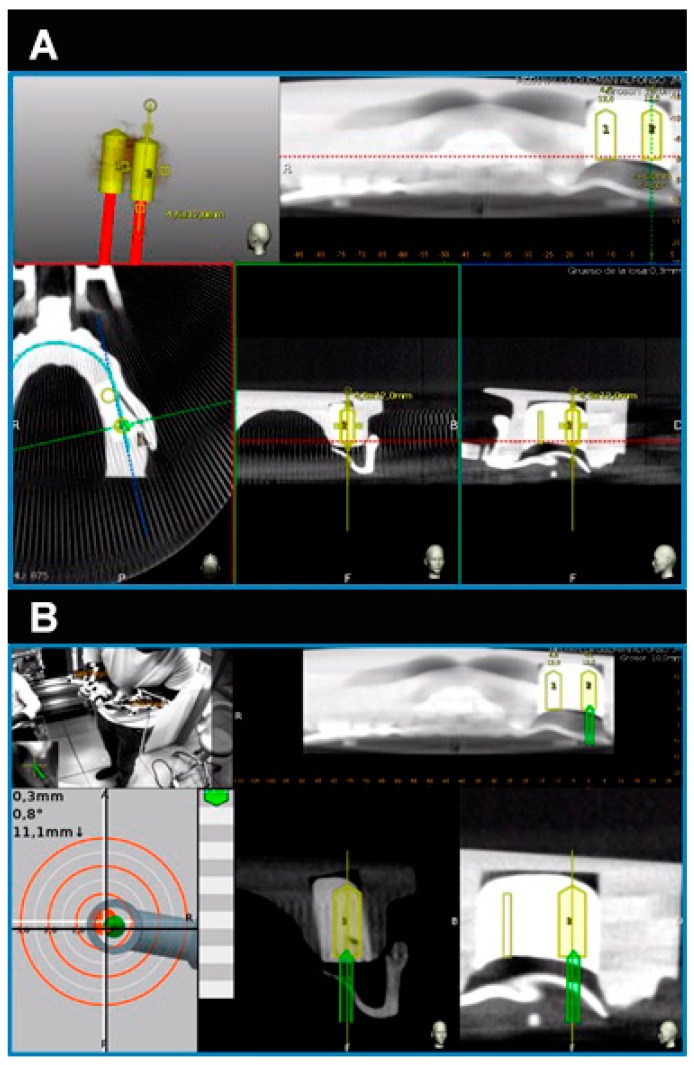
(**A**) Dental implant planning as seen in the treatment-planning software of the computer-aided dynamic navigation system (yellow cylinders) and (**B**) dental implant placement (green cylinders) controlled at all planes and depths.

The NI jaw models were submitted to a preoperative CBCT scan prior to placement of the thermoplastic template (Navistent, ClaroNav, Toronto, ON, Canada) that includes a radiographic marker and an attached handle with a black and white jaw tag, which was fixed over the dental surface of the teeth. CBCT datasets were imported into the treatment-planning software on a mounted laptop computer in a mobile unit (Navident, ClaroNav, Toronto, Canada) to simulate dental implant placement (Figure 2A). Another black and white drill tag was attached to the handpiece. Both optical reference markers were calibrated and recognized by the optical triangulation tracking system composed of stereoscopic motion-tracking cameras which guided the drilling process at the planned angle, pathway, and depth in real time. Socket drilling was performed with a drill (Ref. TSD2041, BioHorizons) and was monitored using the laptop computer containing the computer-aided dynamic navigation system (Figure 2B).

### 2.3. Measurement Procedure

**Figure 3:**
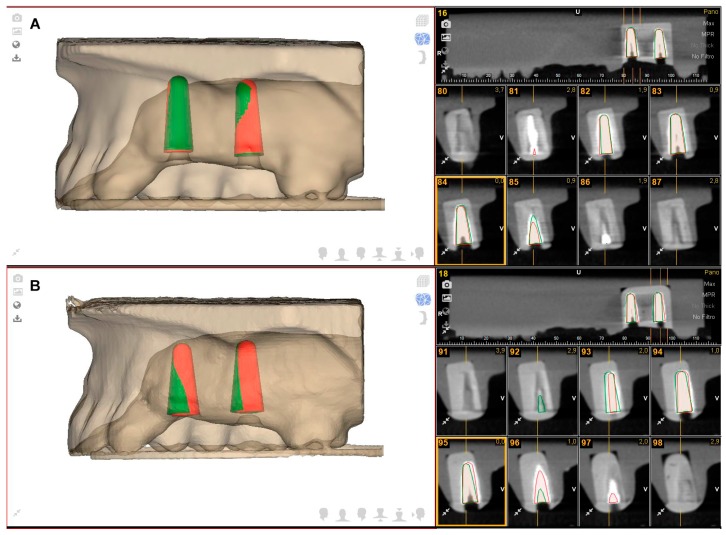
(**A**) Dental implant planning (green cylinders) and dental implant placement (red cylinders) using the computer-aided static navigation system and (**B**) the computer-aided dynamic navigation system.

Following the dental implant placement, postoperative CBCT scans were taken. Dental implant planning and postoperative CBCT scans of the study groups were uploaded into 3D implant-planning software (NemoScan, Nemotec, Madrid, Spain) and matched to analyze apical deviation, which was measured at the apical endpoint; coronal deviation, which was measured at the entry point; and angular deviation, which was measured in the center of the cylinder. All noted deviations of all of the implants were evaluated and compared in the axial, sagittal, and coronal views (Figure 3A,B) using the same expertise operator. Results were expressed according to each position.

### 2.4. Statistical Analysis

Statistical analysis of all variables was carried out using SAS 9.4 (SAS Institute Inc., Cary, NC, USA). Descriptive statistics were expressed as mean and standard deviation (SD) values for quantitative variables and as absolute numbers and percentages for qualitative variables. Comparative analysis was performed by comparing the mean deviation values between planned and performed implant placements using Student’s *t*-test as the variables had a normal distribution. The statistical significance was set at *p* ˂ 0.05.

## 3. Results

**Figure 4:**
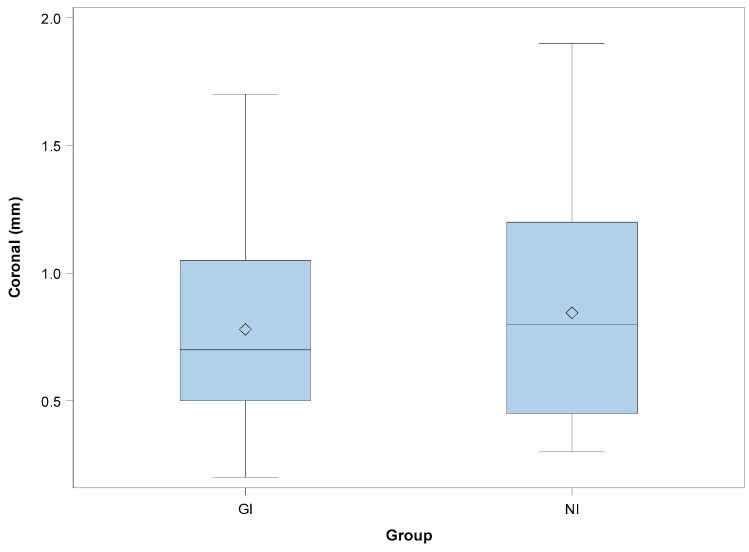
Box plots of the coronal deviations observed in the experimental groups. The horizontal lines in each box represent the median values (GI: static navigation system; NI: dynamic navigation system).

Table 1 shows the mean and SD values. Mean deviations of 0.78 ± 0.43 mm (min: 0.20 mm; max: 1.70 mm) and 0.85 ± 0.48 mm (min: 0.30 mm; max: 1.90 mm) were observed at the coronal entry point of the GI (Figure 3A) and NI (Figure 3B) study groups, respectively (Figure 4).

**Figure 5:**
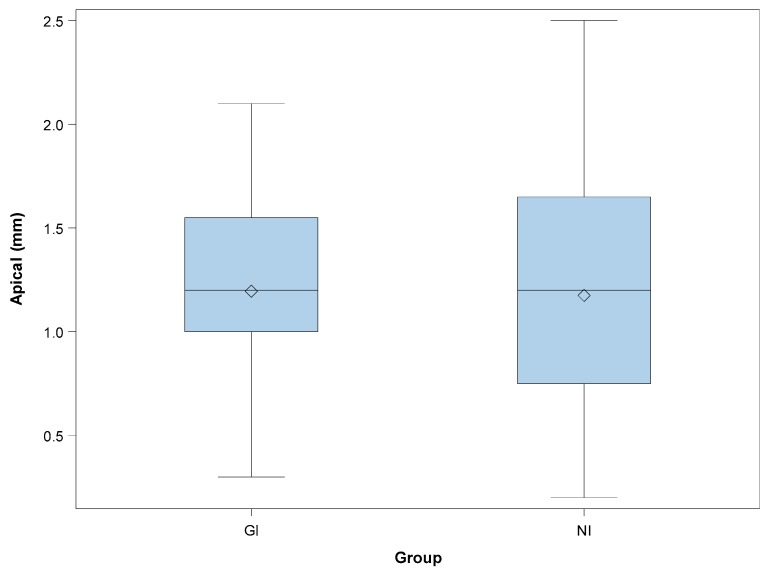
Box plots of the apical deviations observed in the experimental groups. The horizontal lines in each box represent the median values (GI: static navigation system; NI: dynamic navigation system).

The paired *t*-test revealed no statistically significant differences between the coronal deviations of GI and NI (*p* = 0.6535). Mean deviations of 1.20 ± 0.48 mm (min: 0.30 mm; max: 2.10 mm) and 1.18 ± 0.60 mm (min: 0.20 mm; max: 2.50 mm) were observed at the apical endpoint of the GI (Figure 3A) and NI (Figure 3B) study groups, respectively (Figure 5).

**Figure 6:**
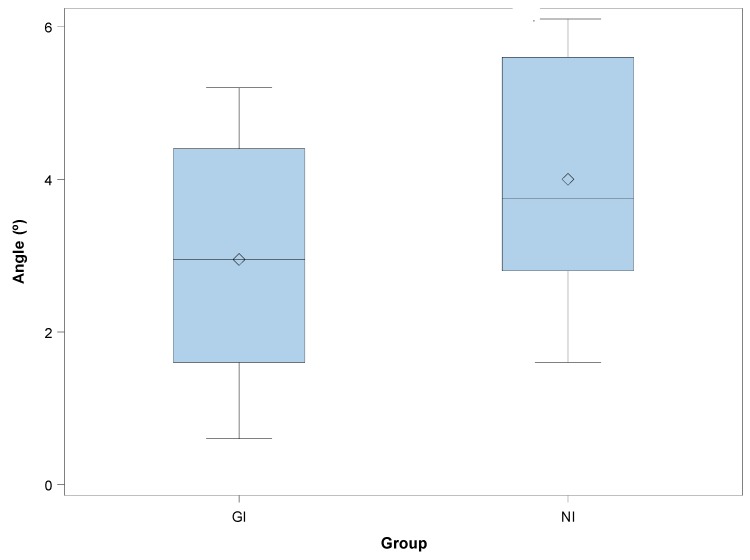
Box plots of the angular deviations observed in the experimental groups. The horizontal lines in each box represent the median values (GI: static navigation system; NI: dynamic navigation system).

The paired *t*-test revealed no statistically significant differences between the apical deviations of GI and NI *(p =* 0.9081). Mean deviations of 2.95° ± 1.48° (min: 0.60°; max: 5.20°) and 4.00° ± 1.41° (min: 1.60°; max: 6.10°) were observed in the GI (Figure 3A) and NI (Figure 3B) study groups, respectively (Figure 6). The paired *t*-test revealed statistically significant differences between the angular deviations of GI and NI *(p =* 0.0272).

## 4. Discussion

The results obtained in the present study rejected the null hypothesis (H0) that stated that there would be no difference between computer-aided static and dynamic navigation systems with regard to the accuracy of dental implant placement. This in vitro study has potential limits related to the precision and stability of the surgical template due to the use of models, which may have significantly differed from what is encountered in a real clinical situation. In recent literature, the accuracy of surgical computer-aided navigation techniques has been widely evaluated and compared. Hoffmann et al. [9] reported statistically significant differences in the accuracy of implant placement between computer-aided dynamic navigation systems and manual implant placement, with mean angular deviations of 4.2 ± 1.8° and 11.2 ± 5°, respectively. Although the methods and selection criteria were slightly different from those used in the present study, implant placement using a computer-aided dynamic navigation system offered a greater degree of accuracy than manual implant placement. Chang-Kai et al. [10] reported similar mean horizontal deviation values at the apical endpoint when using a computer-aided dynamic navigation system (1.35 ± 0.55 mm), a computer-aided static navigation system (1.50 ± 0.79 mm), and manual implant placement (2 ± 0.79 mm). Higher angular deviation values were reported for the computer-aided dynamic navigation system (4.45 ± 1.97°), the computer-aided static navigation system (6.02 ± 3.71°), and manual implant placement (9.26 ± 3.62°). The accuracy of implant placement with computer-aided navigation systems was shown to be better than the degree of accuracy during manual procedures, but the angular deviation values were different from the present findings. The learning requirements of computer-aided dynamic navigation systems might explain the differences between static and dynamic navigation systems [8]. Computer-aided static navigation techniques performed using surgical templates prevent the need for drilling guidance during surgery [1,3,4,5]. Therefore, implant placement accuracy depends directly on the design and manufacturing process of the surgical template; if there is an inaccuracy during the fabrication process, this might lead to intraoperative complications [3]. On the other hand, computer-aided dynamic navigation systems allow for a direct view of the surgical field and provide clinicians with the ability to relocalize the position of an implant, if necessary [9,10]. In addition, these systems are particularly useful in cases of limited mouth openings or treatments in the posterior region [1,8]. The main disadvantage of computer-aided dynamic navigation systems is the difficulty in keeping sight of the dynamic navigation system display during the surgical procedure. However, augmented reality devices could be used to display the virtual image of the computer-aided dynamic navigation system without losing sight of the surgical field [11,12]. Image-guided navigation systems demonstrated comparable accuracy rates regarding control of the depth, position, and angle of implants, which is necessary to avoid intraoperative surgical complications and poor positioning of implants, thereby compromising primary stability and immediate-loading restoration techniques [8,9,12]. In addition, they also avoid the wide excisions often needed to expose the implant platform after the healing period, and enable a minimally invasive transgingival approach to implant placement [1,12]. This is especially helpful in high-risk patients, such as cardiovascular patients taking anticoagulation medications or patients with edentulous, atrophic mandibles [12].

This study shows that computer-aided static and dynamic navigation procedures allow accurate dental implant placement. Nevertheless, further research is needed to determine the influence of the dental implant placement procedure on the accuracy of dental implant positioning and potential clinical complications.

## 5. Conclusions

In conclusion, within the limitations of this study, the results showed that computer-aided static and dynamic navigation procedures allow accurate implant placement. Nevertheless, it is mandatory to perform a CBCT scan and a 3D surface scan and to plan implant placement using specialized surgical planning software. Clinical trials are necessary to analyze the real behavior of these computer-aided navigation procedures.

## Figures and Tables

**Table 1:** Descriptive deviation values at the apical (mm), coronal (mm), and angular (°) levels (guided implant (GI): static navigation system; navigation implant (NI): dynamic navigation system).

	*n*		Mean	SD	Minimum	Maximum
CORONAL	GI	20	0.78	0.43	0.20	1.70
	NI	20	0.85	0.48	0.30	1.90
APICAL	GI	20	1.20	0.48	0.30	2.10
	NI	20	1.18	0.60	0.20	2.50
ANGULAR	GI	20	2.95	1.48	0.60	5.20 *
	NI	20	4.00	1.41	1.60	6.10

## References

[1] Kaewsiri D., Panmekiate S., Subbalekha K., Mattheos N., Pimkhaokham A. (2019). The accuracy of static vs. dynamic computer-assited implant surgery in single tooth space: A randomized controlled trial. Clin. Oral Implants Res..

[2] Herklotz I., Beuer F., Kunz A., Hildebrand D., Happe A. (2017). Navigation in implantology. Int. J. Comput. Dent..

[3] Widmann G., Bale R.J. (2006). Accuracy in Computer-Aided Implant Surgery—A review. Int. J. Oral Maxillofac. Implants.

[4] Chasioti E., Sayed M., Drew H. (2015). Novel Techniques with the Aid of a Staged CBCT Guided Surgical Protocol. Case. Rep. Dent..

[5] Lal K., White G.S., Morea D.N., Wright R.F. (2006). Use of stereolithographic templates for surgical and prosthodontic implant planning and placement. Part II. A clinical report. J. Prosthodont..

[6] Jorba-García A., Figueiredo R., González-Barnadas A., Camps-Font O., Valmaseda-Castellón E. (2019). Accuracy and the role of experience in dynamic computer guided dental implant surgery: An in-vitro study. Med. Oral Patol. Oral Cir. Bucal.

[7] Tahmaseb A., Wu V., Wismeijer D., Coucke W., Evans C. (2018). The accuracy of static computer-aided implant surgery: A systematic review and meta-analysis. Clin. Oral Implants Res..

[8] Stefanelli L.V., DeGroot B.S., Lipton D.I., Mandelaris G.A. (2019). Accuracy of a Dynamic Dental Implant Navigation System in a Private Practice. Int. J. Oral Maxillofac. Implants.

[9] Hoffmann J., Westendorff C., Gomez-Roman G., Reinert S. (2005). Accuracy of navigation- guided socket drilling before implant installation compared to the conventional free-hand method in a synthetic edentulous lower jaw model. Clin. Oral Implants Res..

[10] Chen C.K., Yuh D.Y., Huang R.Y., Fu E., Tsai C.F., Chiang C.Y. (2018). Accuracy of implant placement with a navigation system, a laboratory guide, and freehand drilling. Int. J. Oral Maxillofac. Implants.

[11] Pellegrino G., Mangano C., Mangano R., Ferri A., Taraschi V., Marchetti C. (2019). Augmented reality for dental implantology: A pilot clinical report of two cases. BMC Oral Health.

[12] Gargallo-Albiol J., Barootchi S., Salomó-Coll O., Wang H. (2019). Advantages and disadvantages of implant navigation surgery. A systematic review. Ann. Anat..

